# Health, well-being, and environmental working conditions in the face of COVID-19: sustainable development goal three

**DOI:** 10.1590/0034-7167-2025-0035

**Published:** 2026-07-17

**Authors:** Donizete Vago Daher, Maria Helena Mendonça de Araújo, Irma da Silva Brito, Enéas Rangel Teixeira, Geilsa Soraia Cavalcanti Valente, Fabiana Ferreira Koopmans, Andressa Ambrosino Pinto, Leandro Lourenço da Silva

**Affiliations:** I Universidade Federal Fluminense. Niterói, Rio de Janeiro, Brazil; II Universidade Federal do Amapá. Macapá, Amapá, Brazil; III Universidade de Coimbra. Coimbra, Portugal; IV Universidade do Estado do Rio de Janeiro. Rio de Janeiro, Rio de Janeiro, Brazil; V Universidade Federal do Rio de Janeiro. Macaé. Macaé, Rio de Janeiro, Brazil

**Keywords:** Health, Psychological Well-Being, COVID-19, Sustainable Development, Working Conditions., Salud, Bienestar Psicológico, COVID-19, Desarrollo Sostenible, Condiciones de Trabajo.

## Abstract

**Objectives:**

to analyze healthcare workers’ health and well-being, as well as the environmental conditions of hospital settings during the COVID-19 pandemic, considering the challenge of meeting goal three of the Sustainable Development Goals.

**Methods:**

a descriptive, exploratory, and qualitative study was conducted with 32 healthcare workers employed in a public hospital in Macapá/AP, who contracted COVID-19 and answered interviews using Google Forms^®^ between October and November 2022. Data were organized using IRaMuTeQ^®^ and analyzed using content analysis.

**Results:**

regarding health and well-being, work overload, absenteeism, presenteeism, fear, anxiety, exposure to death, and suffering were reported. Concerning environmental conditions, physical and structural limitations, improvised work environments, and lack of personal protective equipment were reported.

**Final Considerations:**

the need to promote workers’ physical and mental health, improve hospital settings, and reorient work dynamics, especially during times of crisis, minimizing impacts on workers’ health and well-being, was evident.

## RESUMEN

**Objetivos:**

analizar la salud y el bienestar del personal sanitario, así como las condiciones ambientales del entorno hospitalario durante la pandemia de COVID-19, considerando el reto de alcanzar el Objetivo 3 de los Objetivos de Desarrollo Sostenible.

**Métodos:**

estudio descriptivo, exploratorio y cualitativo, con 32 trabajadores sanitarios de hospital público de Macapá/AP, quienes contrajeron COVID-19 y respondieron entrevistas mediante Google Forms^®^ entre octubre y noviembre de 2022. Los datos se organizaron con el IRaMuTeQ^®^ y se analizaron mediante análisis de contenido.

**Resultados:**

sobre la salud y el bienestar, se reportaron sobrecarga laboral, absentismo, presentismo, miedo, ansiedad, exposición a la muerte y sufrimiento. Sobre las condiciones ambientales, se reportaron limitaciones físicas y estructurales, entornos de trabajo improvisados y escasez de equipo de protección personal.

**Consideraciones Finales:**

se evidenció la necesidad de promover la salud física y mental de los trabajadores, mejorar el entorno hospitalario y reorientar la dinámica laboral, especialmente en tiempos de crisis, minimizando los impactos en la salud y el bienestar de los trabajadores.

## INTRODUCTION

The global public health crisis caused by the COVID-19 pandemic between 2020 and 2021 disrupted the implementation of the 17 Sustainable Development Goals (SDGs) defined by the member countries of the United Nations in 2015. The initial goal was to respond to the call to eradicate poverty and protect the planet by 2030 so that there would be peace and prosperity for people, resulting from social, economic, and environmental sustainability^(1)^.

The COVID-19 pandemic, caused by SARS-CoV-2, has represented the most impactful social event on a global scale in recent decades, substantially affecting healthcare systems and necessitating the reorganization of services, the expansion of hospital capacity, the availability of equipment, and material and human resources. Furthermore, it has subjected hospital workers to unprecedented pressure, particularly on their physical and mental health^(2,3)^.

This situation was equally challenging for the whole of Brazil, being exacerbated in several northern states, such as Amapá, which faced difficulties related to its geographical location, limitations regarding available resources, the precariousness of hospital settings, as well as the commodification and devaluation of healthcare workers’ labor activities, especially nurses^(4)^.

Meanwhile, healthcare workers in hospitals were exposed to multiple risk factors due to the demands imposed by combating the pandemic. Measures to promote these workers’ health and well-being, historically undervalued, were relegated to a secondary position as priority was given to saving lives^(5)^.

Several studies have shown an increase in mental health problems, such as fear, anxiety, depression, absenteeism, presenteeism, insomnia, and post-traumatic stress, among workers who performed direct care activities in hospital settings. Work overload and exposure to the virus resulted in significant negative consequences for these workers’ physical and mental health^(5-7)^.

Factors that contributed to this scenario include the lack of material and human resources, constant exposure to death and suffering, uncertainty about their own risk of contagion, and the fear of bringing the virus home and infecting family members^(8)^. In addition, physical illness was another relevant concern, since workers were in direct contact with patients affected by the coronavirus and, consequently, exposed to a greater risk of contracting the disease^(9)^.

The lack, scarcity, or prolonged use of personal protective equipment (PPE), coupled with the low availability of training and/or Continuing Health Education (CHE), has increased workers’ susceptibility to infections and other health problems. Furthermore, grueling work schedules and the absence of sufficient rest periods have also contributed to the weakening of the immune system, further exposing healthcare workers to physical illness^(10)^.

Thus, the individual and collective healthcare practices prevalent in daily hospital work required adaptations and reconfigurations due to the COVID-19 pandemic. In this regard, it became evident that professional practices and new *habitus* were gradually cultivated and incorporated. In this movement, “the incorporation of new *habitus* occurs in response to social conditions and symbolic exchanges within a specific field, reflecting the need for adaptation and adjustment of individual practices to new social demands”, as argued by sociologist Pierre Félix Bourdieu^(11)^.

Faced with the pandemic situation, other *habitus* were unconsciously and naturally incorporated and reproduced, such as the use of gloves, masks, constant hand washing, and the need for donning/doffing PPE to be able to work directly with COVID-19 patients. In this way, a unique professional *habitus* emerged, as well as a movement to produce a different institutional culture, not transmitted through prior guidelines^(12,13)^.

All these transformations demanded a remarkable capacity for adaptation and flexibility from healthcare workers, i.e., the production of a new *habitus*, since many needed to take new responsibilities and face extremely challenging working conditions never before experienced. Given these challenges, the reflections presented in this study are urgent, as they contribute to minimizing knowledge gaps in hospital settings regarding this topic and also align with the SDGs, especially SDG 3^(12,14)^.

## OBJECTIVES

To analyze healthcare workers’ health and well-being, as well as the environmental conditions of hospital settings during the COVID-19 pandemic, considering the challenge of meeting SDG 3.

## METHODS

### Ethical aspects

This study is a product of a thesis defended in 2024 within the Academic Program of Health Care Sciences at the *Universidade Federal Fluminense*. It was approved by the Institute of Scientific and Technological Research of the State of Amapá Research Ethics Committee. All ethical precepts for research with human beings were respected, and participants’ identification data were coded with the letter W (worker), followed by a number (1, 2, 3,... 32) corresponding to their interview, safeguarding anonymity. Participants digitally signed the Informed Consent Form, agreeing to the study.

### Study design

This is a descriptive, exploratory, and qualitative study, relevant for analyzing the health and safety of workers who worked during the COVID-19 pandemic, generating impacts on the reporting practices of occupational health problems, which were previously underreported. Data were grounded in Pierre Félix Bourdieu’s theory, which seeks to understand the *habitus* (behaviors and conceptions of health and well-being) of healthcare workers and hospital environmental conditions. To guide methodological validity, the COnsolidated criteria for REporting Qualitative research was used^(15)^.

### Study setting

The chosen setting was the Occupational Health and Safety Center (In Portuguese, *Núcleo de Saúde e Segurança do Trabalhador* - NSST) of a state-run public hospital, a reference center in the Amazon region, located in Macapá, Amapá state. NSST stands out as the only space accessible to healthcare workers for treatment and reporting of their work-related health problems. During the pandemic, this center’s activities multiplied due to the significant number of workers who became physically and mentally ill as a result of work overload. The hospital, where NSST is located, is extremely important as it is a hospital complex that receives patients from all states in northern Brazil and various riverside communities.

### Participants and inclusion and exclusion criteria

From an initial sample of 215 workers affected by COVID-19 and treated at NSST during 2020 and 2021, 32 of them participated in the interviews sent via Google Forms^®^. This sample recurrently reflected the work overload and presenteeism of the institution’s workers, who often remained on call uninterruptedly.

Healthcare workers (nurses, physiotherapists, physicians, pharmacists, psychologists, and nursing and laboratory technicians) treated at NSST, with a confirmed COVID-19 test result, who signed the informed consent form and answered the interview were included. Those who were on vacation, leave, or absent due to comorbidities and advanced age (risk for COVID-19) were excluded.

### Data collection and organization

Data collection was carried out through interviews during October and November 2022, considered by the WHO to still be a pandemic period^(16)^. The questionnaire was administered via the Google Forms^®^ platform to 32 healthcare workers who answered the following questions: how did professional behaviors or practices (individual and collective) manifest themselves during the COVID-19 period? What was the role of management/support team in the face of the possibility of becoming ill with COVID-19? Did work relationships and occupational risks in the workplace influence workers’ health and well-being?

For data organization, the *Interface de R pour les Analyses Multidimensionnelles de Textes et de Questionnaires* (IRaMuTeQ^*®*^) software was used, which is based on the R software and allows for different types of analyses, such as Descending Hierarchical Classification, similarity analysis (SA), word cloud, among others^(17,18)^.

SA proved to be the most suitable method for explaining the phenomenon studied, as it allowed for the identification of co-occurrences between words and the clear visualization of connectivity relationships between vocabulary, enabling similarities to be grouped into colored halos^(17,18)^.

The text *corpus* was constituted by all the responses returned from the 32 participants. Using software, 48 text segments were extracted from the general text, representing the analyzed excerpts. The processed corpus had a 100% utilization of Elementary Context Units, thus considered extremely representative^(18)^.

### Data analysis

Initially, the interviews were transcribed, the data was coded, and the guiding topics of participants’ thinking were identified, thus fulfilling the stages of pre-analysis, material exploration, and interpretative synthesis of results, according to Bardin’s content analysis technique^(19)^.

## RESULTS

Data analysis revealed the following composition among the professional categories as follows: 14 nurses (43.8%); five nursing technicians (15.6%); five physicians (15.6%); three laboratory technicians (9.4%); three physiotherapists (9.4%); one pharmacist (3.1%); and one psychologist (3.1%). The age range spanned from 30 to 61 years, and they worked in both outpatient clinics and inpatient units.

With the statements, topics were produced that will be analyzed below, seeking to meet the objective and the questions chosen for the study.

### Professional behaviors, *habitus*, and management role in the context of COVID-19

**Figure 1:**
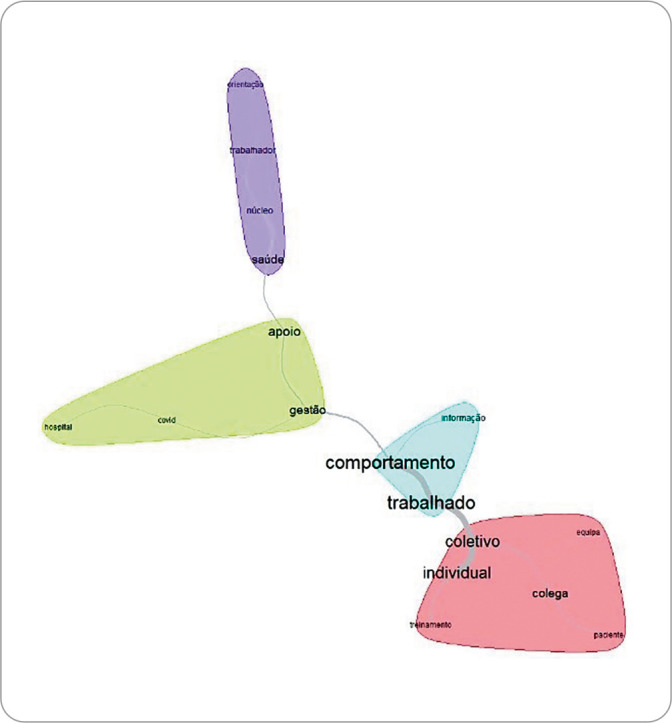
Behaviors, work relationships, and management in the context of COVID-19

SA generated four halos with a direct relationship between the words “behavior”, “management”, “collective”, “individual”, and “health” (Figure 1).

Healthcare workers reported that certain behaviors were produced from the possibility of exposure to and illness from the coronavirus (SARS-CoV-2). These new behaviors are known as *habitus*. It was evident that these behaviors resulted from the lack of training and/or CHE on how to deal with COVID-19 and how to care for oneself, as well as long working hours and the existence of more than one job among the workers at the institution analyzed. These conditions further increased professional stress and the possibility of direct or indirect contamination due to work overload and the inability to cope with the new biosafety demands raised by the pandemic.

Other factors that contributed to the increased exposure included reduced work team sizes due to sick co-workers, absenteeism, presenteeism, absences due to comorbidities, and the sharing of common spaces such as rest areas and cafeterias. They also indicated individual and collective behaviors of disregard for biosafety standards. Thus, the workers reported:

*Contact with other co-workers in meetings, discussions of clinical cases and procedures, or even during meals and in the few free hours available, may have contributed to the illness. Regarding the fragility of health, I believe that physical exhaustion, long working hours, lack of professionals* [who took time off when they became ill], *and excessive demands were important factors.* (W8)

*I realized that many professionals at the hospital, like myself, did not receive adequate support from management, as no training plans, interpersonal relationship strengthening programs, or follow-up programs were conceived or implemented.* (W22)

Individual and collective professional behaviors, i.e., *habitus* in this pandemic work context, have also emerged as a factor of exposure through contact among co-workers in various encounters, being identified as one of the possible modes of transmission of the COVID-19 virus.

It is worth highlighting that work in a hospital setting is carried out by multidisciplinary teams, operating in multiple sectors across different areas of activity, with diverse employment relationships and shifts, working collectively, both within and among teams.

### Interfaces between work team, work environment, health and well-being: a challenge for Sustainable Development Goal 3

In this context, the SA generated from the statements produced five halos, with a prevalence of the words “patient”, “risk”, “PPE”, “team”, “nursing”, “well-being”, “being” and “COVID-19”.

Healthcare teams in hospital settings, especially nursing staff who carried out their daily activities during the pandemic, were even more exposed to different types of occupational risks. In this reality, according to the findings, these professionals’ health and well-being became invisible both to managers and to other co-workers, and even to society.

Participants’ statements were recurrent, mainly citing precarious infrastructure to care for a large number of patients, lack of PPE for all workers, who often had to purchase their own PPE, and even a lack of water for their own consumption, among others.

**Figure 2:**
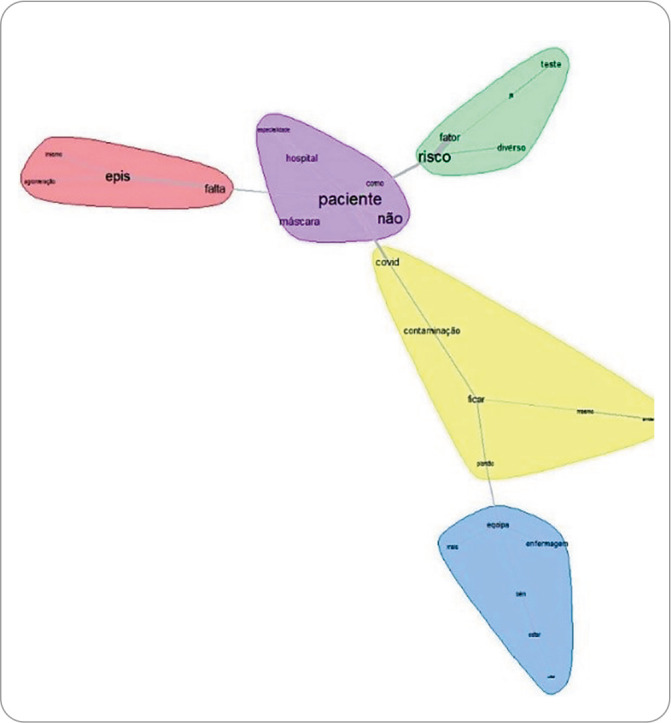
Work team, work environment, health, and well-being

Figure 2 presents a set of words that express the major challenges to containing COVID-19 among healthcare workers and indicates actions that should promote their health and well-being, in line with what is advocated by SDG 3 (Patient, no, mask, hospital, risk, COVID, contamination).

It is also noteworthy that the words “mask” and “lack”, being further apart within the halo, may represent a failure in supply or the absence of this PPE, especially during the beginning of the pandemic. There were mentions in the reports that workers should acquire their own PPE.

*The lack of materials and supplies for the work, coupled with precarious physical environments, needed improvement because they exposed workers to risks and hindered the team’s performance.* (W21)

*The lack or absence of essential materials made it difficult to carry out most activities and stressed the professional. This situation was aggravated during the pandemic, where we often had to buy masks, gloves, caps, gowns, and even 70% alcohol.* (W25)

Hence, during the pandemic, the lack of infrastructure, supplies, PPE, COVID-19 tests, environmental sanitation, among other things, increased the likelihood of exposure and transmission of the virus among healthcare workers, compromising the quality and safety of care provided, as well as these professionals’ health, well-being, and lives.

To prevent COVID-19 in the workplace, it was found, through direct observation and the consulted literature, that healthcare workers should adopt biosafety, epidemiological surveillance, and health protection measures, such as case monitoring, contact tracing, isolation of suspected and confirmed cases, periodic testing, correct use of PPE, hand hygiene, social distancing, among others.

## DISCUSSION

The COVID-19 pandemic has reshaped healthcare workers’ behaviors and care practices. This new reality resulted in the reduction or suspension of routine inpatient unit services, prioritizing the numerous cases of COVID-19 patients, thus physically and mentally overburdening workers. Consequently, these changes further weakened workers’ health and well-being who were on the front lines of the pandemic response^(1,20)^.

To understand the discontinuity and regression in the implementation process of the 17 SDGs, as well as the importance of operationalizing SDG 3, it is essential to reflect on healthcare workers’ statements regarding the COVID-19 pandemic, its impact on healthcare systems and these professionals’ lives, as well as the educational, infrastructural, and organizational challenges faced by all those who carried out their practices in hospital settings.

The qualitative, descriptive, and exploratory approach was essential to analyze healthcare workers’ behaviors, practices, and *habitus*, as well as their experiences and perceptions during the COVID-19 pandemic, especially the challenges faced by professionals dealing with adverse, unprecedented circumstances that produced significant levels of physical and emotional stress. In this sense, Bourdieu contributes to this study by arguing that a new *habitus* is not acquired through imposition, but rather through the free incorporation of lived and shared experiences^(13,21)^.

IRaMuTeQ^®^ was used as a tool to explore, spatialize, filter, and organize the concept words which, combined with Bardin’s content analysis, contributed to understanding the set of statements and generating categories that expressed participants’ thinking^(17-19,22)^.

National and international studies have repeatedly shown that healthcare workers in higher-risk/vulnerable areas have had their professional *habitus* reshaped: longer working hours; new demands related to their dual employment; inadequate use of PPE (due to lack of or poor training); and failure to adopt biosafety standards. This new *habitus* thus represented a greater likelihood of infection by the virus^(4,23-25)^.

Regarding training and/or CHE actions, it became evident that these should be coordinated between NSST and hospital management, with effective communication and attention to demands. However, the culture of not offering these actions and the reorganization of the work environment and professional behaviors during the pandemic deteriorated the environmental working conditions, reaffirming the postponement of SDG 3^(14,26)^.

However, different scholars on this same topic indicate that other behaviors related to teamwork can prevail, even in times of crisis, pointing to these as positive, such as effective communication, cooperation, and mutual support. These factors, when adopted by healthcare institutions, could contribute to other forms of professional practices for the prevention of COVID-19 in the workplace, as well as for the promotion of workers’ health and well-being. In this movement, possibilities for concrete actions to promote health and well-being could be opened, aiming at SDG 3^(27,28)^.

With regard to healthcare teams, it is noteworthy that nursing teams were even more exposed to different occupational risks (biological, physical, chemical, ergonomic, accident, and psychosocial stressors)^(29)^ produced by close and prolonged contact with patients at various stages of the disease.

Among the numerous factors cited by participants that worsened the work environment during COVID-19, the following stood out: absence or scarcity of supply and distribution of PPE, precarious hospital infrastructure, improvised beds, scarcity of hospital materials and supplies, as well as lack of potable water for personal use. These emerged as real risk factors for exposure and impairment to workers’ physical and mental health and well-being^(20)^.

The use of PPE, recommended in biosafety standards as one of the protective measures against exposure to the coronavirus, has become mandatory in the routine of all healthcare professionals. Studies have revealed that healthcare workers must use PPE according to the risk level of each activity, following the indications, methods of use, conservation, and disposal, as governed by the technical standards and clinical protocols established by the competent bodies^(30,31)^.

To reduce the circulation of the virus, studies have shown that it is necessary to take measures to interrupt the chain of transmission, as well as to strengthen and improve the response capacity of healthcare systems, thus ensuring workers’ safety and health^(20,32)^. These actions would contribute significantly to strengthening health promotion and preventing new or recurring cases, maintaining each worker’s health and well-being.

Nevertheless, healthcare workers who served on the front lines of the COVID-19 pandemic also experienced the need to balance professional responsibilities with personal and family demands. The fear of carrying the virus home and infecting loved ones caused additional distress, especially if there were older adults and/or people with comorbidities in that family context who could further expose them to illness and the subsequent risk of serious COVID-19 complications. These factors substantially increased psychological stress, fear, anxiety, presenteeism, and the risk of burnout^(33-35)^.

This, coupled with feelings of loss and a sense of professional powerlessness, has led, in many cases, to recurring physical and mental exhaustion, affecting healthcare workers’ ability to continue providing quality care and, consequently, increasing absenteeism. Notably, SDG 3, regarding self-management of care, has been significantly worsened, with many cases of compromised physical and mental health^(24,36,37)^.

It has therefore become evident that, with regard to SDG 3, the need for investment in actions to promote health and prevent disease remains relevant, placing healthcare workers, and especially nursing professionals, with work autonomy, in salutogenic environments, “ensuring healthy lives and promote well-being for all at all ages”^(38,39)^.

### Study limitations

A limitation of this study is that it was conducted in a single NSST at a public hospital in Macapá. This limited the participation of a larger number of healthcare workers affected by COVID-19. Another limitation is the mandatory remote interview process, due to the social isolation measures recommended by the Brazilian Ministry of Health.

### Contributions to nursing, health, or public policy

In 2020 and 2021, many studies on COVID-19 were published in national and international journals, as it is an emerging pathology. However, using the SDG correlation, healthcare workers and COVID-19, and considering SDG 3, the number of scientific publications is reduced. Thus, this study may contribute to reviews and interventions in certain hospital units with similar contexts, aiming at workers’ health and well-being and, consequently, improving the effectiveness and care provided to patients and their families.

## FINAL CONSIDERATIONS

This study aimed to analyze the health, well-being, and working conditions experienced by healthcare workers during the COVID-19 pandemic from the perspective of SDG 3 (Good Health and Well-Being). This aim was achieved by highlighting, through statements, behaviors, *habitus*, and environmental conditions from Bourdieu’s perspective.

The results indicated that healthcare workers, especially those in nursing teams, experienced high levels of stress, fear, and anxiety, as well as being exposed to various contamination factors, due to work overload, structural limitations, lack of PPE, and low availability of training and/or CHE to work with the unique challenges of COVID-19.

It is important to highlight the need for training and/or CHE strategies to minimize the after-effects of this condition, now called post-COVID-19 syndrome, as well as the clinical picture of burnout syndrome, especially among healthcare workers who worked on the front lines of the fight against the pandemic. To this end, it is necessary to implement new NSSTs to promote and protect workers’ physical and mental health. In this regard, improving working conditions and the infrastructure of healthcare systems in Brazil would break the cycle of discontinuity in SDG 3 implementation.

## Data Availability

The research data are available only upon request.
