# Supplementary material for: Health, well-being, and environmental working conditions in the face of COVID-19: sustainable development goal three
Source: Rev Bras Enferm. 2026 Jul 17;79:e20250035. doi: 10.1590/0034-7167-2025-0035 (PMC13379197; doi:10.1590/0034-7167-2025-0035)
Supplement: Translation

# Translation: Saúde, bem-estar e condições ambientais de trabalho na COVID-19: objetivo três do desenvolvimento sustentável

**DOI:** 10.1590/0034-7167-2025-0035pt

**Authors:** Donizete Vago Daher, Maria Helena Mendonça de Araújo, Irma da Silva Brito, Enéas Rangel Teixeira, Geilsa Soraia Cavalcanti Valente, Fabiana Ferreira Koopmans, Andressa Ambrosino Pinto, Leandro Lourenço da Silva

**Affiliations:** I Universidade Federal Fluminense. Niterói, Rio de Janeiro, Brasil; II Universidade Federal do Amapá. Macapá, Amapá, Brasil; III Universidade de Coimbra. Coimbra, Portugal; IV Universidade do Estado do Rio de Janeiro. Rio de Janeiro, Rio de Janeiro, Brasil; V Universidade Federal do Rio de Janeiro. Macaé. Macaé, Rio de Janeiro, Brasil

**Keywords:** Saúde, Bem-Estar Psicológico, COVID-19, Objetivos de Desenvolvimento Sustentável, Ambiente de Trabalho.

## Abstract

**Objetivos:**

analisar a saúde e o bem-estar dos trabalhadores de saúde, bem como as condições ambientais do contexto hospitalar na pandemia de COVID-19, considerando o desafio de atender à meta três dos Objetivos de Desenvolvimento Sustentável.

**Métodos:**

estudo descritivo, exploratório e qualitativo, com 32 trabalhadores de saúde atuantes em hospital público de Macapá/AP, acometidos por COVID-19, que responderam entrevistas pelo *Google Forms* ^®^ entre outubro e novembro de 2022. Dados foram organizados pelo *software* IRaMuTeQ^®^ e analisados pela análise de conteúdo.

**Resultados:**

sobre saúde e bem-estar, relataram-se sobrecarga de trabalho, absenteísmo, presenteísmo, medo, ansiedade, exposição à morte e sofrimento. Sobre as condições ambientais, relataram-se limitações físico-estruturais, improvisação de ambiente e escassez de equipamentos de proteção individual.

**Considerações Finais:**

evidenciou-se necessidade de promover saúde física e mental dos trabalhadores, melhoria da ambiência hospitalar, e reorientação das dinâmicas laborais, especialmente para tempos de crise, minimizando impactos sobre a saúde e bem-estar de trabalhadores.

## INTRODUÇÃO

A crise global na saúde pública, ocasionada pela pandemia de COVID-19, entre 2020 e 2021, descontinuou a implementação dos 17 Objetivos de Desenvolvimento Sustentável (ODS), definidos pelos países membros da Organização das Nações Unidas em 2015. A meta inicial seria responder ao apelo para erradicar a pobreza e proteger o planeta, até 2030, para que houvesse paz e prosperidade para as pessoas, em decorrência da sustentabilidade social, econômica e ambiental^(1)^.

A pandemia de COVID-19, originada pelo SARS-CoV-2, representou, nas últimas décadas, o evento de maior impacto social em escala global, afetando, substancialmente, os sistemas de saúde, impondo a necessidade de reorganização dos serviços, a ampliação da capacidade hospitalar, da disponibilidade de equipamentos, e de recursos materiais e humanos. Ainda, determinou a sujeição de trabalhadores da área hospitalar a uma pressão inédita, com destaque para a saúde física e mental^(2,3)^.

Essa situação foi, igualmente, desafiadora para todo o Brasil, sendo exacerbada em vários estados do norte do país, como o estado do Amapá, que enfrentou dificuldades relativas à sua localização geográfica, às limitações referentes aos recursos disponíveis, à precarização da ambiência hospitalar, bem como ao processo de mercantilização e desvalorização das atividades laborais dos trabalhadores de saúde, especialmente de enfermagem^(4)^.

Nesse ínterim, os trabalhadores de saúde da área hospitalar foram expostos a múltiplos fatores de riscos em função das exigências impostas pelo combate à pandemia. As medidas de promoção da saúde e de bem-estar desses trabalhadores, historicamente subvalorizadas, foram secundarizadas, na medida em que se priorizava o atendimento de vidas em risco^(5)^.

Diferentes estudos evidenciaram o aumento de problemas mentais, como medo, ansiedade, depressão, absenteísmo, presenteísmo, insônia e estresse pós-traumático nos trabalhadores que exerciam atividades voltadas ao cuidado direto no contexto hospitalar. A sobrecarga de trabalho e a exposição ao vírus resultaram em significativas consequências negativas para a saúde física e mental desses trabalhadores^(5-7)^.

Fatores que contribuíram para esse cenário incluem a escassez de recursos materiais e humanos, a exposição constante à morte e ao sofrimento, a incerteza quanto ao próprio risco de contágio, e o medo de levar o vírus para seus domicílios, infectando familiares^(8)^. Além disso, o adoecimento físico foi outra preocupação relevante, visto que os trabalhadores estiveram em contato direto com pacientes acometidos pelo coronavírus e, consequentemente, expostos a um maior risco de contrair a doença^(9)^.

A falta, escassez ou, ainda, o uso prolongado de equipamentos de proteção individual (EPIs), acrescida da baixa oferta de ações de capacitação e/ou de Educação Permanente em Saúde (EPS), aumentou a suscetibilidade dos trabalhadores a infecções e outros problemas de saúde. Ademais, jornadas extenuantes e a ausência de períodos suficientes de descanso também contribuíram para o enfraquecimento do sistema imunológico, expondo, ainda mais, os trabalhadores de saúde ao adoecimento físico^(10)^.

Assim, as práticas individuais e coletivas de assistência à saúde vigentes nos cotidianos de trabalho hospitalar necessitaram de adaptações e reconfigurações, devido ao momento pandêmico da COVID-19. Nesse sentido, ficou evidenciado que práticas profissionais e novos *habitus* foram, aos poucos, cultivados e incorporados. Nesse movimento, “a incorporação de novos *habitus* ocorre em resposta às condições sociais e às trocas simbólicas dentro de um campo específico, refletindo a necessidade de adaptação e ajuste das práticas individuais às novas exigências sociais”, como defende o sociólogo Pierre Félix Bourdieu^(11)^.

Diante da situação pandêmica, outros *habitus* foram sendo inconscientemente e, naturalmente, incorporados e reproduzidos, como uso de luvas, máscaras, lavagem constante das mãos e a necessidade de (des)paramentação para poder atuar diretamente com os pacientes com COVID-19. Experienciava-se e vivenciava-se, desta forma, o surgimento de um *habitus* profissional singular, bem como de um movimento de produção de uma outra cultura institucional, não retransmitida por meio de orientações prévias^(12,13)^.

Todas essas transformações demandaram dos trabalhadores de saúde uma notável capacidade de adaptação e flexibilidade, ou seja, de produção de um novo *habitus,* uma vez que muitos precisaram assumir novas responsabilidades e enfrentar condições laborais extremamente desafiadoras nunca antes vivenciadas. Diante desses desafios, são prementes as reflexões que tomam lugar neste estudo, uma vez que as mesmas contribuem minimizando as lacunas do conhecimento no cenário hospitalar referente a esta temática, indo, também, ao encontro dos ODS, especialmente o ODS 3^(12,14)^.

## OBJETIVOS

Analisar a saúde e o bem-estar dos trabalhadores de saúde, bem como as condições ambientais do contexto hospitalar na pandemia de COVID-19, considerando o desafio de atender à meta três dos ODS.

## MÉTODOS

### Aspectos éticos

Este estudo é um produto gerado por uma tese, defendida em 2024, junto ao Programa Acadêmico de Ciências do Cuidado em Saúde da Universidade Federal Fluminense. Foi aprovado pelo Comitê de Ética em Pesquisa do Instituto de Pesquisas Científicas e Tecnológicas do Estado do Amapá. Todos os preceitos éticos para pesquisa com seres humanos foram respeitados, e os dados de identificação dos participantes foram codificados pela letra T (trabalhador), seguido de número (1, 2, 3,...32) correspondente à sua entrevista, resguardando anonimato. Os participantes assinaram, digitalmente, o Termo de Consentimento Livre e Esclarecido (TCLE), concordando com o estudo.

### Tipo de estudo

Trata-se de estudo descritivo, exploratório e qualitativo, relevante por analisar a saúde e segurança dos trabalhadores que atuaram durante a pandemia de COVID-19, gerando impactos nas práticas de notificação dos agravos laborais, até então, subnotificados. Dados foram ancorados na teoria de Pierre Félix Bourdieu, que busca compreender o *habitus* (comportamentos e concepções de saúde e bem-estar) dos trabalhadores de saúde e as condições ambientais hospitalares. Para nortear a validade metodológica, utilizou-se o instrumento *COnsolidated criteria for REporting Qualitative research* ^(15)^.

### Cenário do estudo

Foi eleito como cenário o Núcleo de Saúde e Segurança do Trabalhador (NSST) de um hospital público, estadual, de referência na região amazônica, localizado em Macapá, estado do Amapá. O NSST se destaca como o único espaço a ser acessado pelos trabalhadores de saúde para fins de atendimentos e notificações de seus agravos laborais. Durante o período pandêmico, este núcleo teve as suas atividades multiplicadas pelo expressivo número de trabalhadores adoecidos física e mentalmente diante da sobrecarga de trabalho. O hospital, espaço onde encontra-se o NSST, é de extrema relevância por ser um complexo hospitalar que recebe pacientes de todos os estados da região Norte do Brasil e diferentes regiões ribeirinhas.

### Participantes e critérios de inclusão e exclusão

De uma amostra inicial de 215 trabalhadores acometidos por COVID-19 e atendidos no NSST, durante os anos de 2020 e 2021, 32 deles participaram da entrevista enviadas através do *Google Forms* ^®^. Essa amostra refletiu, de modo recorrente, a sobrecarga de trabalho e o presenteísmo dos trabalhadores da instituição, que muitas vezes permaneciam nas escalas de plantão de forma ininterrupta.

Foram incluídos trabalhadores de saúde (enfermeiros, fisioterapeutas, médicos, farmacêuticos, psicólogos, e técnicos de enfermagem e de laboratório) atendidos no NSST, com resultado de exame confirmado para COVID-19, que assinaram o TCLE e responderam à entrevista. Foram excluídos os que estavam de férias, licença ou afastados por comorbidades e idade elevada (risco para COVID-19).

### Coleta e organização dos dados

A coleta de dados foi realizada por meio de entrevistas, durante os meses de outubro e novembro de 2022, considerado pela OMS ainda como período pandêmico^(16)^. O formulário foi aplicado via plataforma *Google Forms* ^®^ aos 32 trabalhadores de saúde que responderam às seguintes questões: como se concretizaram os comportamentos ou práticas profissionais (individuais e coletivas) durante o período da COVID-19? Qual o papel da gestão/equipe de apoio diante da possibilidade de adoecimento com COVID-19? As relações de trabalho e os riscos ocupacionais no ambiente laboral influenciaram a saúde e o bem-estar dos trabalhadores?

Para organização dos dados, foi utilizado o *software Interface de R pour les Analyses Multidimensionnelles de Textes et de Questionnaires* (IRaMuTeQ^®^), que se ancora no *software* R, que permite diferentes tipos de análises, como Classificação Hierárquica Descendente, análise de similitude (AS), nuvem de palavra, entre outras^(17,18)^.

A AS demonstrou ser a mais indicada para explicar o fenômeno estudado, pois permitiu identificar as coocorrências entre as palavras e visualizar, de forma clara, as relações de conectividade entre os vocábulos, possibilitando que semelhanças pudessem ser reunidas em halos coloridos^(17,18)^.

O *corpus* textual foi constituído pela totalidade das respostas retornadas dos 32 participantes. Por meio do *software*, foram extraídos, do texto geral, 48 segmentos de texto, representando os recortes analisados. O *corpus* processado teve aproveitamento de 100% de Unidades de Contexto Elementar, considerado, assim, extremamente representativo^(18)^.

### Análise de dados

Inicialmente, houve a transcrição das entrevistas, a codificação de dados e a identificação das temáticas norteadoras do pensamento dos participantes, atendendo, assim, às etapas de pré-análise, exploração do material e síntese interpretativa dos resultados, de acordo com a técnica de análise de conteúdo de Bardin^(19)^.

## RESULTADOS

A análise dos dados evidenciou a seguinte composição entre as categorias profissionais: 14 enfermeiros (43,8%); cinco técnicos de enfermagem (15,6%); cinco médicos (15,6%); três técnicos de laboratório (9,4%); três fisioterapeutas (9,4%); um farmacêutico (3,1%); e um psicólogo (3,1%). A faixa etária abrangeu entre 30 e 61 anos, e esses laboravam tanto em ambulatórios quanto em unidades de internação.

Com os depoimentos, foram produzidas temáticas que serão analisadas a seguir, buscando atender ao objetivo e às questões eleitas para o estudo.

### Comportamentos profissionais, *habitus* e o papel da gestão no contexto da COVID-19

A AS gerou quatro halos com relação direta entre as palavras “comportamento”, “gestão”, “coletivo”, “individual” e “saúde” (Figura 1).

**Figura 1:** Comportamentos, relações de trabalho e gestão no contexto da COVID-19

Os trabalhadores de saúde relataram que determinados comportamentos foram produzidos a partir da possibilidade da exposição e adoecimento pelo coronavírus (SARS-CoV-2). Esses novos comportamentos são os *habitus.* Evidenciou-se que esses fatos foram decorrentes da não oferta de capacitação e/ou EPS sobre como lidar com a COVID-19 e como cuidar de si, assim como dos longos turnos de trabalho e existência de mais de um vínculo empregatício dos trabalhadores da instituição analisada. Essas condições aumentaram, ainda mais, o estresse profissional e a possibilidade de contaminação direta ou indireta pela sobrecarga de trabalho, e incapacidade de lidar com as novas demandas de biossegurança suscitadas pela pandemia.

Outros fatores que contribuíram para o aumento da exposição incluíram a redução do tamanho das equipes de trabalho devido a colegas que adoeceram, absenteísmo, presenteísmo, ausências devido a comorbidades e o compartilhamento de espaços comuns, como áreas de descanso e refeitório. Sinalizaram, ainda, comportamentos individuais e coletivos de descaso quanto às normas de biossegurança. Assim, os trabalhadores reportaram:

*O contato com outros colegas em reuniões, discussão de casos clínicos, condutas, ou ainda durante alimentação e nas poucas horas vagas podem ter facilitado o adoecimento. Para a fragilidade da saúde, acho que o cansaço físico, as longas horas trabalhadas, escassez de profissionais* [que se afastavam ao adoecer] *e o excesso de demandas foram fatores importantes*. (T8)

*Percebi que muitos profissionais no hospital, assim como eu, não receberam apoio adequado da gestão, pois nenhum plano de capacitação, de reforço nos relacionamentos interpessoais e de acompanhamento foram pensados e implementados.* (T22)

Os comportamentos profissionais individuais e coletivos, ou seja, o *habitus* neste contexto pandêmico de trabalho também se configuraram como fator de exposição pelo contato entre colegas em encontros diversos, sendo apontados como um dos modos possíveis de transmissão do vírus da COVID-19.

Ressalta-se que o trabalho no contexto hospitalar é desenvolvido por equipes multiprofissionais, com atuação em múltiplos setores de diferentes áreas de atividades, com vínculos e turnos diversos, atuando, de forma coletiva, inter e entre equipes.

### Interfaces entre equipe de trabalho, ambiente laboral, saúde e bem-estar: desafio para o Objetivo de Desenvolvimento Sustentável 3

Nesta temática, a AS gerada a partir dos depoimentos produziu cinco halos, com prevalência das palavras “paciente”, “risco”, “EPI”, “equipe”, “enfermagem”, “bem”, “estar” e “COVID-19”.

As equipes de trabalho em saúde no contexto hospitalar, especialmente as da enfermagem que desenvolveram suas atividades cotidianas no período pandêmico, ficaram ainda mais expostas aos diferentes tipos de riscos ocupacionais. Nessa realidade, a saúde e o bem-estar desses profissionais, segundo os achados, passaram a ser invisibilizados tanto por parte de gestores quanto por parte de outros colegas de trabalho e, até mesmo, pela sociedade.

Os depoimentos dos participantes foram recorrentes, citando, principalmente, precária infraestrutura para atender um grande número de pacientes, escassez de EPIs para todos os trabalhadores, que muitas vezes precisaram adquirir os seus próprios EPIs, e até mesmo falta de água para o consumo próprio, entre outros.

A Figura 2 apresenta um conjunto de palavras que expressam os grandes desafios para contenção da COVID-19 entre os trabalhadores de saúde e indicações de ações que deveriam promover a saúde e bem-estar dos mesmos, indo ao encontro do que preconiza o ODS 3.

**Figura 2:** Equipe de trabalho, ambiente laboral, saúde e bem-estar

Destaca-se, ainda, que as palavras “máscara” e “falta”, estando mais distantes no interior do halo, podem representar a falha no abastecimento ou a ausência desse EPI, especialmente durante o início da pandemia. Houve menções, nos relatos, de que os trabalhadores deveriam adquirir seus próprios EPIs.

*A falta de materiais e insumos para o trabalho, com ambientes físicos precários, precisariam melhorar porque expunham os trabalhadores a riscos e dificultavam o bom desempenho da equipe.* (T21)

*A carência ou ausência de materiais essenciais dificultava a execução da maioria das atividades e estressava o profissional. Fato esse agravado na pandemia, onde muitas vezes tivemos que comprar máscaras, luvas, gorro, capotes e até mesmo o álcool 70%.* (T25)

Neste sentido, durante a pandemia, a falta de infraestrutura, de insumos, de EPI, de testes de COVID-19, de higienização de ambientes, entre outros, aumentou a probabilidade de exposição e de transmissão do vírus entre os trabalhadores de saúde, comprometendo a qualidade e segurança da assistência prestada, bem como a saúde, o bem-estar e a vida desses profissionais.

Para prevenir a COVID-19 no ambiente de trabalho, constatou-se, por meio de observação direta e pela literatura consultada, que os trabalhadores de saúde deveriam adotar medidas de biossegurança, de vigilância epidemiológica e de proteção à saúde, como monitoramento dos casos, rastreamento de contatos, afastamento dos suspeitos e confirmados, testagem periódica, uso correto dos EPIs, higienização das mãos, distanciamento social, entre outras.

## DISCUSSÃO

A pandemia de COVID-19 reconfigurou comportamentos e práticas assistenciais realizadas pelos trabalhadores de saúde. Essa nova realidade resultou na redução ou suspensão de atendimentos rotineiros das unidades de internação, priorizando os inúmeros atendimentos aos pacientes com COVID-19, sobrecarregando física e mentalmente os trabalhadores. Logo, essas mudanças fragilizaram, ainda mais, a saúde e o bem-estar dos trabalhadores que atuavam na linha de frente no enfrentamento à pandemia^(1,20)^.

Para se compreender a descontinuidade e o retrocesso do processo de implementação dos 17 ODS, bem como a importância da operacionalização do ODS 3, é primordial fazer uma reflexão a partir de depoimentos de trabalhadores de saúde sobre o período pandêmico da COVID-19, seu impacto nos sistemas de saúde, na vida desses profissionais, assim como sobre os desafios educativos, infraestruturais e organizacionais a serem enfrentados por todos que desenvolviam suas práticas no contexto hospitalar.

A abordagem qualitativa, descritiva e exploratória foi essencial para analisar os comportamentos, as práticas e o *habitus* de trabalhadores de saúde, além de suas experiências e percepções durante a pandemia de COVID-19, especialmente os desafios enfrentados pelos profissionais que lidavam em circunstâncias adversas, sem precedentes e produtoras de significativos quadros de estresse físico e emocional. Nesse sentido, Bourdieu contribui com este estudo defendendo que um novo *habitus* não é apreendido pela imposição, mas sim pela incorporação livre de experiências vividas e compartilhadas^(13,21)^.

O *software* IRaMuTeQ^®^ foi utilizado como uma ferramenta para explorar, espacializar, filtrar e organizar as palavras-conceitos que, somadas à análise de conteúdo de Bardin, colaboraram para compreensão do conjunto de depoimentos e geração de categorias que expressaram o pensamento dos participantes^(17-19,22)^.

Estudos nacionais e internacionais evidenciaram, de forma recorrente, que os trabalhadores de saúde que atuavam em áreas de maior risco/vulnerabilidade tiveram seu *habitus* profissional remodelado: jornadas de trabalho mais longas; novas demandas de ações em seus duplos vínculos empregatícios; uso inadequado dos EPIs (devido à ausência ou fragilidade de capacitação); e a não adoção das normas de biossegurança. Esse novo *habitus* representou, assim, maior probabilidade de infecção pelo vírus^(4,23-25)^.

Em relação às ações de capacitação e/ou EPS, ficou evidenciado que elas deveriam ocorrer articuladas entre o NSST e a gestão hospitalar, com comunicação efetiva e atendimento das demandas. Contudo, a cultura da não oferta dessas ações e a reorganização da ambiência e dos comportamentos profissionais na pandemia deterioraram as condições ambientais de trabalho, reafirmando o adiamento do ODS 3^(14,26)^.

Contudo, diferentes estudiosos sobre esta mesma temática sinalizam que podem prevalecer, mesmo em momentos de crise, outros comportamentos referentes ao trabalho em equipe, apontando estes como positivos, como a comunicação efetiva, a cooperação e o apoio mútuo. Esses fatores, aos serem adotados pelas instituições de saúde, poderiam contribuir com outras formas de práticas profissionais para prevenção da COVID-19 no ambiente de trabalho, bem como para promoção da saúde e bem-estar dos trabalhadores. Nesse movimento, poderiam ser abertas possibilidades de concretude às ações de promoção da saúde e bem-estar, objetivando o ODS 3^(27,28)^.

Em relação as equipes de saúde, destaca-se que as de enfermagem ficaram ainda mais expostas aos diferentes riscos ocupacionais (biológicos, físicos, químicos, ergonômicos, de acidentes e estressores psicossociais)^(29)^ produzidos pelo contato próximo e duradouro com os pacientes em diversos estágios da doença.

Entre os inúmeros fatores citados pelos participantes e que agravaram o ambiente laboral durante a COVID-19, destacaram-se ausência ou escassez de oferta e distribuição de EPI, infraestrutura de internação precária, leitos improvisados, escassez de material e insumos hospitalares, assim como falta de água potável para uso pessoal. Esses figuraram como reais fatores de risco de exposição e comprometimento à saúde física, mental e bem-estar dos trabalhadores^(20)^.

O uso dos EPIs, recomendados nas normas de biossegurança como uma das medidas protetivas contra exposição ao coronavírus, passou a compor, de forma obrigatória, a rotina de todos os profissionais de saúde. Estudos revelaram que os trabalhadores de saúde devem usar os EPIs de acordo com o nível de risco de cada atividade, seguindo as indicações, as formas de uso, de conservação e de descarte, conforme regem as normas técnicas e os protocolos clínicos estabelecidos pelos órgãos competentes^(30,31)^.

Para reduzir a circulação do vírus, estudos evidenciaram que se faz necessário tomar medidas para interrupção da cadeia de transmissão, assim como fortalecer e qualificar a capacidade de resposta dos sistemas de saúde, garantindo, desse modo, segurança e saúde aos trabalhadores^(20,32)^. Essas ações contribuiriam, sobremaneira, para o fortalecimento da promoção da saúde e prevenção de novos ou reincidentes agravos, mantendo a saúde e o bem-estar de cada trabalhador.

Não obstante, trabalhadores de saúde que atuaram na linha de frente no enfretamento da pandemia de COVID-19, igualmente, experienciaram a necessidade de conciliar responsabilidades profissionais com demandas pessoais e familiares. O receio de carrear o vírus para seus domicílios e infectar entes queridos causou angústia adicional, especialmente se nesse contexto familiar havia idosos e/ou pessoas com comorbidades que poderiam expor, ainda, ao adoecimento e posterior risco de complicações graves da COVID-19. Esses fatos elevaram, substancialmente, o estresse psicológico, medo, ansiedade, presenteísmo e ao risco de *burnout* ^(33-35)^.

Isso, somado ao sentimento de perda e a sensação de impotência profissional, levou, em muitos casos, ao recorrente esgotamento físico e mental, afetando a capacidade dos trabalhadores de saúde de continuar prestando cuidados de qualidade e, consequentemente, aumentando o absenteísmo. Notoriamente, o ODS 3, em relação à autogestão do cuidado, foi totalmente agravado, com muitos casos de comprometimento da saúde física e mental^(24,36,37)^.

Tornou-se evidente, portanto, que, em relação ao ODS 3, continua atual a necessidade de investimentos em ações de promoção da saúde e prevenção de doenças, colocando os trabalhadores de saúde e, principalmente, os de enfermagem, com autonomia de trabalho, em espaços salutogênicos, “assegurando uma vida profissional saudável e promotora de bem-estar para todas e todos, em todas as idades”^(38,39)^.

### Limitações do estudo

Aponta-se como limitação este estudo ter sido desenvolvido em um único NSST do hospital público de Macapá, que dispunha de serviço ocupacional. Esse fato limitou a participação de um maior número de trabalhadores de saúde acometidos por COVID-19. Considera-se, também, como outra limitação a obrigatoriedade da realização das entrevistas de modo remoto, motivado pelo isolamento social preconizado pelo Ministério da Saúde, Brasil.

### Contribuições para as áreas da enfermagem, saúde ou políticas públicas

Em 2020 e 2021, muitos estudos sobre a COVID-19 foram publicados em periódicos nacionais e internacionais, por se tratar de uma patologia emergente. Contudo, utilizando-se a correlação ODS, trabalhadores de saúde e COVID-19, e considerando-se a o ODS 3, as publicações científicas são em número reduzido. Assim, este estudo poderá contribuir com revisões e intervenções em determinadas unidades hospitalares de contextos semelhantes, visando à saúde e ao bem-estar dos trabalhadores e, consequentemente, melhoria da efetividade e assistência prestada aos pacientes e familiares.

## CONSIDERAÇÕES FINAIS

A proposta deste estudo foi a de analisar a saúde, o bem-estar e as condições dos ambientes laborais vivenciados por trabalhadores de saúde, durante a pandemia de COVID-19, na perspectiva do ODS 3 (Saúde e Bem-Estar). A proposta foi contemplada na medida em que singularizou, a partir de depoimentos, os comportamentos, *habitus* e condições ambientais na perspectiva bourdieusiana.

Os resultados apontaram que os trabalhadores de saúde, principalmente os das equipes de enfermagem, vivenciaram altos níveis de estresse, medo e ansiedade, assim como foram expostos a diversos fatores de contaminação, em decorrência da sobrecarga de trabalho, limitações estruturais, escassez de EPI e baixa oferta de capacitação e/ou EPS para trabalhar com a singularidade da COVID-19.

Assinala-se como relevante a oferta de estratégias de capacitação e/ou EPS que minimizem as sequelas desse agravo hoje denominada síndrome pós-COVID-19, bem como do quadro clínico da síndrome de *burnout*, especialmente daqueles trabalhadores de saúde que atuaram na linha de frente do combate à pandemia. Para tal, faz-se necessário a implementação de novos NSSTs, com o propósito de promover e proteger a saúde física e mental dos trabalhadores. Nesse sentido, melhorar as condições de trabalho e de infraestrutura dos sistemas de saúde no Brasil romperia o processo de descontinuidade de implementação do ODS 3.

## Data Availability

Os dados de pesquisa só estão disponíveis mediante solicitação.
